# Editorial: Signaling by primary cilia in development and disease

**DOI:** 10.3389/fcell.2023.1186367

**Published:** 2023-03-21

**Authors:** Sung-Eun Kim, Inna Nechipurenko, Søren Tvorup Christensen

**Affiliations:** ^1^ Department of Pediatrics, Dell Pediatric Research Institute, Dell Medical School, The University of Texas at Austin, Austin, TX, United States; ^2^ Department of Biology and Biotechnology, Worcester Polytechnic Institute, Worcester, MA, United States; ^3^ Department of Biology, University of Copenhagen, Copenhagen, Denmark

**Keywords:** primary cilia, signaling pathways, ciliogenesis, ciliopathy, ciliary genes

Primary cilia are antenna-like cell structures that protrude in a single copy from the surface of most vertebrate cell types to detect extracellular cues that regulate embryonic patterning, organogenesis, and adult tissue/organ homeostasis. The capacity of primary cilia to detect and transduce signaling information into cellular processes relies on their unique complement of receptors, ion channels, and associated signal transduction modules, e.g., G protein-coupled receptor (GPCR), receptor tyrosine kinase (RTK), Hedgehog, Wnt, transforming growth factor-beta (TGFB)/bone morphogenetic protein (BMP), and extracellular matrix (ECM) signaling (Wheway et al., 2018; Anvarian et al., 2019). All cilia are compartmentalized by a transition zone (TZ), a specialized gating structure found at cilia base, that controls the entry and exit of proteins, including receptors, and thereby the signaling properties of the primary cilium. Consequently, mutations in genes that encode TZ components or proteins that regulate ciliary assembly and intraciliary trafficking processes cause a spectrum of more than 35 human diseases and syndromes, collectively called ciliopathies, that are commonly associated with obesity, renal anomalies, neurodevelopmental and psychiatric impairment, craniofacial and skeletal defects and congenital heart disease (Reiter and Leroux, 2017). Our Research Topic comprises the reviews and research articles that examine the function of select ciliary genes and signaling systems during embryonic development, highlight tissue-specific mechanisms in cilia assembly and localization of ciliary signaling proteins, and discuss how defects in these ciliary systems translate into ciliopathies.

Multiple protein complexes are required for assembly and sensory properties of primary cilia. A modified mother centriole called the basal body anchors the cilium to the cell membrane in part *via* distal appendage proteins (DAPs) (Tanos et al., 2013). Removal of the centrosomal protein 110 (CP110) from the distal end of the mother centriole initiates the ciliary assembly. The work from Lee et al. demonstrated the differential role of the DAP, sodium channel and clathrin linker 1 (Sclt1) in ciliogenesis during embryonic fore- and hindlimb development in novel *Sclt1* mutant mice. Sclt1 regulates ciliogenesis in developing hindlimb mesenchymal cells by facilitating recruitment of Ttbk2 and removal of Cp110 from the mother centriole, which is associated with a response to Sonic hedgehog signaling. The authors provide the tissue-specific DAP assembly programs during embryonic limb morphogenesis, suggesting multiple pathogenic functions of ciliary genes in pleiotropic phenotypes of ciliopathies.

Intraflagellar transport (IFT) is a bidirectional transport system that tracks along the polarized microtubules of the axoneme and mediates ciliary assembly, maintenance, and signaling through the transport of ciliary cargo proteins into or out of the organelle (Lechtreck, 2015). Bidirectionality is maintained by anterograde kinesin-2 and retrograde IFT-dynein motors, which in conjunction with IFT-B and IFT-A complexes, respectively, transport cargoes to and from the ciliary tip (Lechtreck, 2022). The BBSome, a complex composed of eight Bardet Biedl syndrome (BBS) proteins, is a membrane cargo adapter that recognizes transmembrane proteins such as receptors in Hedgehog and class A GPCR signaling and links them to the IFT machinery (Lechtreck, 2022). BBS has a broader spectrum of phenotypes, including cognitive impairment and obesity, supporting the critical role of BBS proteins in brain development (Brinckman et al., 2013). The minireview by Zheng et al. overviews the structure and cellular functions of IFT172 in ciliary assembly and maintenance as well as Sonic hedgehog signaling activation. Hedgehog signaling is one of the best-characterized cilia-based signaling systems in vertebrates and one of the critical morphogens regulating tissue patterning and morphogenesis during embryonic development (Riddle et al., 1993; Briscoe and Small, 2015) as well as adult tissue homeostasis and repair (Petrova and Joyner, 2014). Zheng et al. discuss the mutations of IFT172 found in ciliopathies, e.g., Joubert Syndrome (JS), Mainzer-Saldino syndrome (MZSDS) and BBS, and remark on the importance of further investigation into the functional and pathogenic mechanisms of IFT172 in ciliopathies. In line with the function of the BBSome in ciliary receptor trafficking, Stubbs et al. analyze the localization of GPCRs (somatostatin receptor 3 (Sstr3), melanin-concentrating hormone receptor 1 (Mchr1), dopamine receptor 1 (D1), Gpr161, and Gpr19) and their downstream effectors (type 3 adenylyl cyclase and β-arrestin) in different brain regions of a novel *Bbs1* knockout mouse. The authors observe intriguing brain-region-specific differences in ciliary localization of the above GPCR pathways’ components and demonstrate that BBSome may control ciliary entry/retention of Sstr3 and Mchr1 and exit of D1, Gpr161, and Gpr19, thus revealing additional complexity in protein- and cell-type-specific mechanisms guiding ciliary localization of signaling proteins in the developing brain.

Xu and Tang overview the role of cholesterol in regulating multiple aspects of Sonic hedgehog signaling, including posttranslational modification and transportation of proteins in this pathway. The authors further review the reciprocal effects of Sonic hedgehog signaling in cholesterol metabolism and homeostasis and discuss the birth defects, e.g., holoprosencephaly, associated with mutations of Sonic hedgehog genes.

Emerging evidence points to a critical function of primary cilia is detecting morphogenic cues in combination with biomechanical forces, such as through interaction with the extracellular matrix (ECM) to control neuronal differentiation in the developing and adult brain. The minireview by Pittman and Solecki focuses on the pivotal role of primary cilia in balancing the morphogen (e.g., Hedgehog) sensing and integrin-ECM interaction during cerebellar granule neuron (CGN) differentiation. Specifically, the authors discuss how different ECM components influence cilia maintenance in a niche-specific manner during CGN differentiation and bring into focus the intriguing cooperative roles of integrins and cilia in sensing the mechanical forces in the developing cerebellum.

In summary, the articles in this Research Topic enhance the understanding of the ciliary structural components and signaling pathways during development, provide additional insight on the pathogenesis of ciliopathies and birth defects, and bring forth insightful ideas for future investigations.
